# Influence of prokaryotic microorganisms on initial soil formation along a glacier forefield on King George Island, maritime Antarctica

**DOI:** 10.1038/s41598-021-92205-z

**Published:** 2021-06-23

**Authors:** Patryk Krauze, Dirk Wagner, Sizhong Yang, Diogo Spinola, Peter Kühn

**Affiliations:** 1 GFZ, German Research Centre for Geosciences, Helmholtz Centre Potsdam, Section Geomicrobiology, 14473 Potsdam, Germany; 2 Institute of Geosciences, University of Potsdam, 14476 Potsdam, Germany; 3 Department of Geosciences, Research Area Geography, Laboratory of Soil Science and Geoecology, Eberhard Karls University Tübingen, 72070 Tübingen, Germany; 4 Present Address: Department of Chemistry and Biochemistry, University of Alaska Fairbanks, Fairbanks, 99775-6160 USA

**Keywords:** Microbiology, Biogeochemistry

## Abstract

Compared to the 1970s, the edge of the Ecology Glacier on King George Island, maritime Antarctica, is positioned more than 500 m inwards, exposing a large area of new terrain to soil-forming processes and periglacial climate for more than 40 years. To gain information on the state of soil formation and its interplay with microbial activity, three hyperskeletic Cryosols (vegetation cover of 0–80%) deglaciated after 1979 in the foreland of the Ecology Glacier and a Cambic Cryosol (vegetation cover of 100%) distal to the lateral moraine deglaciated before 1956 were investigated by combining soil chemical and microbiological methods. In the upper part of all soils, a decrease in soil pH was observed, but only the Cambic Cryosol showed a clear direction of pedogenic and weathering processes, such as initial silicate weathering indicated by a decreasing Chemical Index of Alteration with depth. Differences in the development of these initial soils could be related to different microbial community compositions and vegetation coverage, despite the short distance among them. We observed—decreasing with depth—the highest bacterial abundances and microbial diversity at vegetated sites. Multiple clusters of abundant amplicon sequence variants were found depending on the site-specific characteristics as well as a distinct shift in the microbial community structure towards more similar communities at soil depths > 10 cm. In the foreland of the Ecology Glacier, the main soil-forming processes on a decadal timescale are acidification and accumulation of soil organic carbon and nitrogen, accompanied by changes in microbial abundances, microbial community compositions, and plant coverage, whereas quantifiable silicate weathering and the formation of pedogenic oxides occur on a centennial to a millennial timescale after deglaciation.

## Introduction

Retreating glaciers in polar and mountainous regions reveal proglacial terrain that is exposed to soil formation and subsequently colonized by microorganisms and plants^1–4^. Considering the particular vulnerability of the Antarctic environment to climate change, studies on soils from glacier forelands could provide indications of how climate changes at the global scale will affect soil formation at the regional scale. By substituting space with time, chronosequences of proglacial environments are an important tool to understand primary succession and soil forming processes^5^, and were therefore used to study the succession of soil microbial communities and their influence on initial soil formation in the past^6–8^. Such microbial populations with different abundances, community structures and diversities are among the first organisms to colonize recently deglaciated areas^9–11^. Their activities within biogeochemical cycles such as the fixation of carbon and nitrogen into bioavailable forms^6,7^ can promote environmental changes that facilitate the succession of organisms at higher trophic levels^12–14^. In order to understand the relationship between primary and secondary succession in proglacial environments and to shed light on the influence of microbial processes on the development of initial soil ecosystems and vice-versa, it is crucial to study the factors that shape the genetic structure of local microbial populations of such environments^13^.

The present microbial communities in ice-free areas of polar regions are dominated by Acidobacteria, Actinobacteria, Bacteroidetes, Firmicutes, Gemmatimonadetes and Proteobacteria^10,15,16^, which are well adapted to their harsh environment^17,18^. These microbial communities have been described to be influenced by local soil chemical parameters, such as pH^19^, and soil physical parameters such as grain size distribution and soil moisture^20^. Also, the microclimate^21^, vegetation cover, and cryoturbation processes^22^ play a role in the observed soil properties (e.g. bulk density, soil temperature) in Antarctica. Thus, these properties and processes could have an impact on soil microbial community composition and activity.

Compared to ice-free areas of continental Antarctica, the soils from maritime Antarctica differ significantly due to higher water availability and warmer temperatures, which lead to deeper active layers and promote vegetative cover and mineral weathering^23–25^. Following the regional warming during the last 50 years, a significant loss of ice volume and melting of many outlet glaciers in maritime Antarctica could be observed^26–28^. The glacial retreat will probably keep its accelerated pace due to the continuous warming over Antarctica by 0.34 °C per decade^29^. It will continuously affect soil-forming processes and microbial activity in maritime Antarctica by exposing glacial sediments^30–32^, which offers an excellent setting to investigate initial soil-forming processes and the colonization by microbial pioneers before higher plants succeed^4,30^. Particularly, the frontal retreat of the Ecology Glacier on King George Island, South Shetland Islands, with approx. 30 m per year since the early nineties opens new terrain for soil-forming processes and terrestrial life^33^. In a centennial to millennial timescale, the carbon and nitrogen content, as well as the pH are the main soil properties to change on King George Island, leading to the formation of soil horizons^34^. Similar findings were observed from glacier forelands in Europe, particularly from the Alps in Switzerland^35–38^. A recent study in the foreland of the Ecology Glacier on King George Island demonstrated that the diversity and properties of microorganisms in recently deglaciated areas are not only related to age but also to differences in soil stability within the upper centimeters due to the influence of cryoturbation^39^. Nevertheless, there is still a deficiency of information about the decadal-scale changes of soil properties and their interplay with microorganisms in soil ecosystems in maritime Antarctica.

We hypothesize that prokaryotic microorganisms initiate/drive soil properties changes (e.g. soil organic carbon accumulation, weathering) within decades after deglaciation. To test our hypothesis, we related the investigated soil properties to microbial community structure and microbial abundances in the foreland deglaciated after 1979 and compared it with an older soil distal to the lateral moraine (deglaciated before 1956) of the Ecology Glacier. To capture the heterogeneity of the soil landscape, three soils in close proximity (maximum distance of 150 m) formed on the same substrate and in a similar topographic position but with differing vegetation cover were sampled. These soils, which represent a recently deglaciated area, were compared with an older soil that had formed on a similar substrate that had been deglaciated before 1956. We combined grain size and pedochemical analyses with DNA-based molecular biological analyses, including high-throughput sequencing and quantitative PCR, to determine the diversity, distribution, and abundance of microbial communities.

## Material and methods

### Study area

King George Island is located in the South Shetland Islands archipelago. The stratigraphy of King George Island comprises Upper Cretaceous to Lower Miocene predominantly subaerial volcanic and volcanoclastic rocks. Fossiliferous marine and glaciomarine sediments are more common in the Oligocene to Lower Miocene rocks. Quaternary volcanism along the southern margin of King George Island and the axial part of Bransfield Strait is related to back-arc extension^40^. The rocks exposed after the frontal retreat of the glacier are mainly mafic volcanic rocks from the Arctowski Cove Formation.

The relatively mild and moist conditions in maritime Antarctica compared to continental Antarctica result in frequent freeze–thaw cycles, which foster periglacial processes (e.g. cryoturbation), and chemical and physical weathering^41^. Moreover, a usually water-saturated active layer during the summer increases biological and chemical weathering and accumulation of organic carbon^42^. As a result, a suite of soil-forming processes occurs on the island, such as cryoturbation, gleization, melanization, paludization, and phosphatization. Since most of the soils are relatively young and weakly developed (~ 4000 year BP since the last deglaciation on King George Island)^43^, these processes are closely linked to the landscape position, parent material and faunal activity (e.g. penguin rookeries). The main resulting soil orders are Cryosols, Leptosols, Cambisols, and Histosols^41,42,44^, but also different soil groups such as Arenosols and Gleysols may occur^45^. Additionally, Podzols, Umbrisols, Stagnosols and Gleysols were found in the surrounding area of the Arctowski Station on King Georges Island^46,47^.

**Figure 1:**
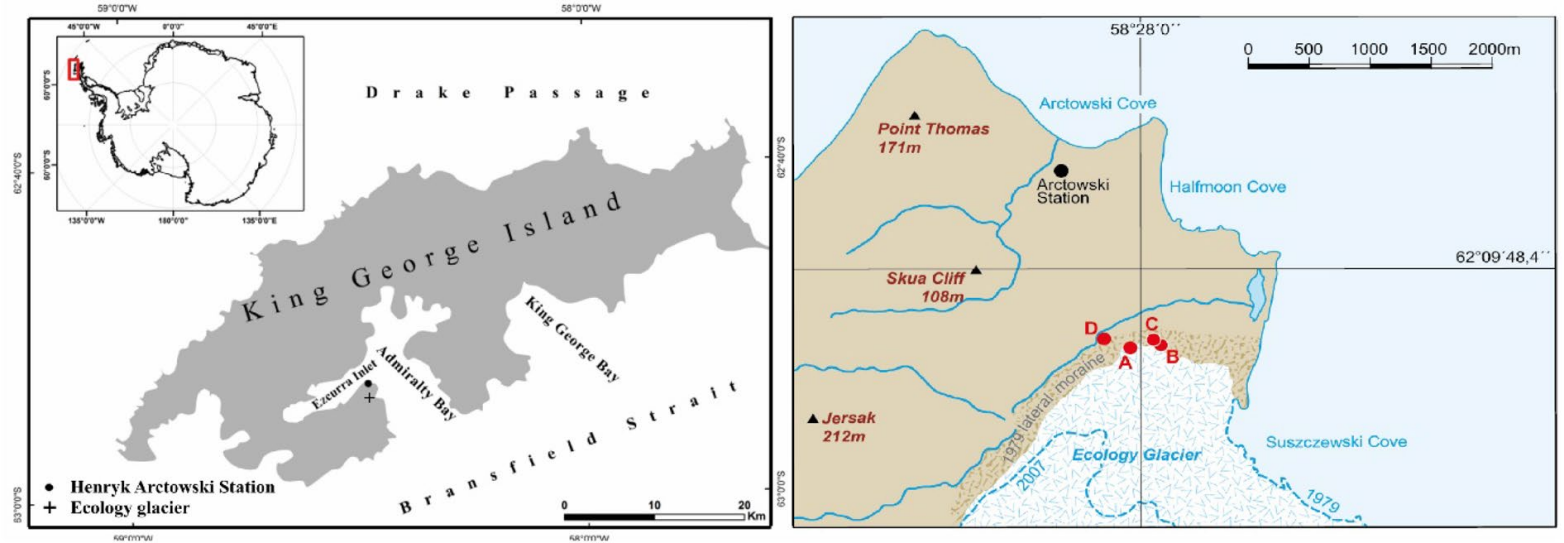
Location of the study sites. Soil profile locations close to the Ecology Glacier are marked as red dots. Soil profiles A, B, and C are located in the glacier foreland deglaciated after 1979^33^. Profile D is located distal to the lateral moraine. The dashed blue lines indicate the glacier front in 1979 and 2007^50^ (source: Orthophotomap from 2007, Department of Antarctic Biology, Polish Acadamy of Sciences).

The study site is located in the foreland of the Ecology Glacier on King George Island characterized by an oceanic polar climate. Temperature measurements recorded by the Chilean Antarctic station President Eduardo Frei Montalva from 1971 to 2004 indicate a mean annual temperature of − 2.3 °C with the coldest temperatures in July and August (mean temperature − 6.5 °C) and the warmest in February (mean temperature 1.6 °C). The mean annual precipitation is < 500 mm, with maximum precipitation during spring/autumn and a minimum during summer/winter^48^. The margin of the Ecology Glacier was > 500 m inward in 2014 compared with the front line during the late 1970s, where the ice reached the sea (Fig. 1). The present coastline represents approximately the front line of summer 1956/1957 after Birkenmajer^33^. After a period of slow deglaciation (4–4.5 m per year) between 1979 and 1989, the speed of deglaciation increased significantly between 1989 and 2001^49^.

**Figure 2:**
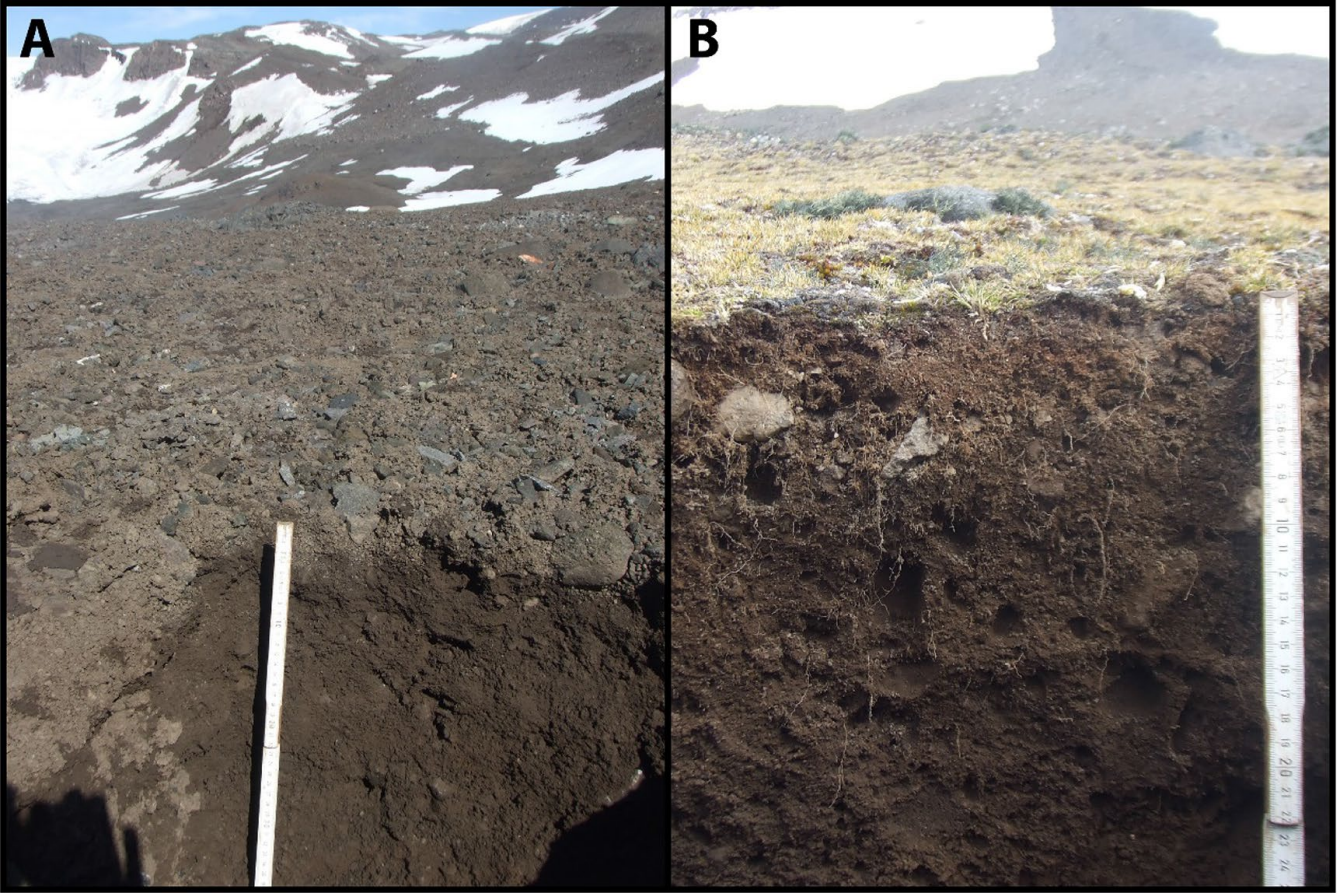
Photographs of the investigated Cryosols on King George Island, South Shetland Islands. (**A**) KGI A, a hyperskeletic Cryosol, was located in the foreland of the Ecology Glacier, which was deglaciated after 1979. (**B**) Soil profile KGI D, a Cambic Cryosol, was located distal to the lateral moraine of the Ecology Glacier and was deglaciated before 1956.

The profiles KGI A, B, and C are located within 150 m distance on the same lateral moraine substrate deglaciated after 1979. These sites are within the sampling zone III of Zdanowski^39^. We sampled three soil profiles (A, B, C) from well-drained positions of lateral moraine deposits with slightly different leeside/windward positions in the present foreland of the Ecology Glacier (Fig. 2).

**Figure 3:**
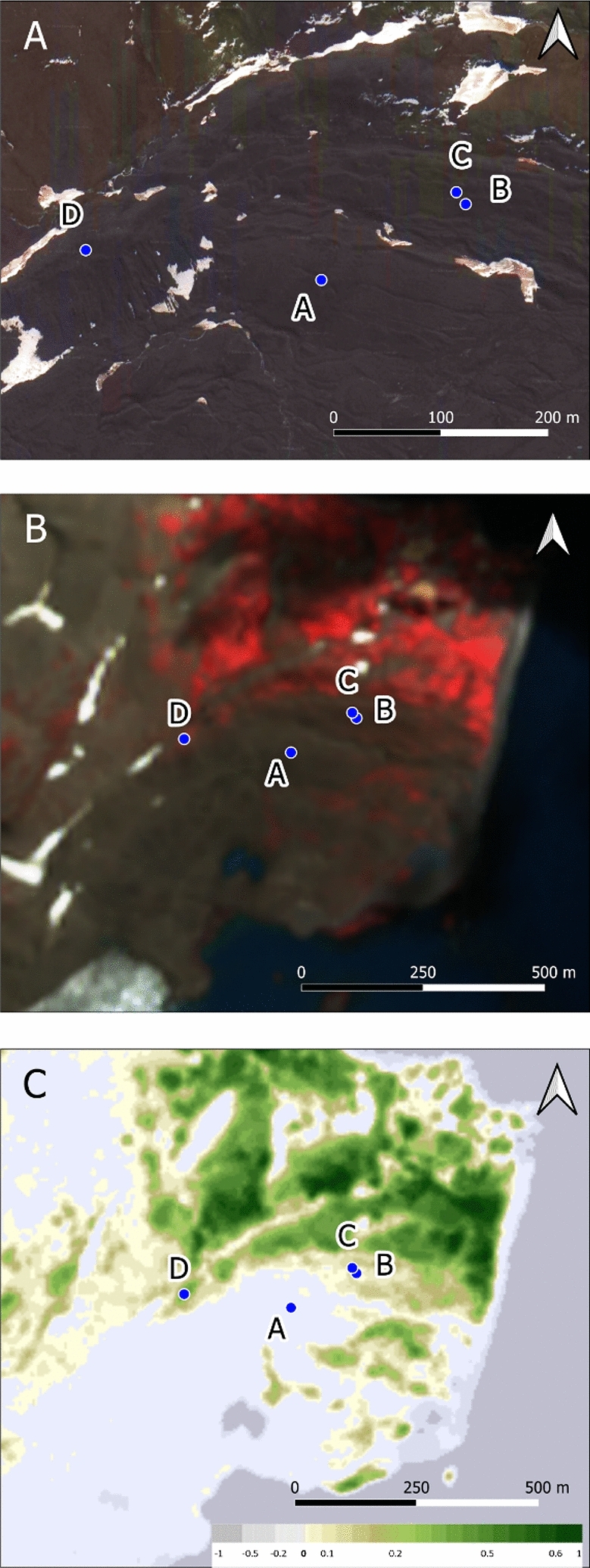
(**A**) High resolution satellite image from 06.11.2016 (Map data^©^ 2016 Google). (**B**) False colour Sentinel-2 image from 19.01.2020 (red = band 8, green = band 4, blue = band 3) for enhanced visualisation of the vegetation. (**C**) Normalized Difference Vegetation Index (NDVI), dimensionless with positive values indicating healthy vegetation and negative values indicating the presence of open water bodies. Contains modified Copernicus data.

One soil profile (KGI D) is located in a well-drained position distal to the lateral moraine (Figs. 1, 3A) on a substrate deglaciated before 1956. The substrate of all profiles is mainly composed of volcanic material from the Arctowski Cove Formation and very similar across all four profiles except for the Ah horizon of KGI D. None of the investigated soil profiles are influenced by penguin or bird rookeries. Vegetation coverage differed between the sample sites (Fig. 3B). KGI A showed no vegetation, whereas KGI B and C showed small amounts of vegetation, and KGI D was fully covered in vegetation. Conversely, chlorophyll values were the highest at KGI D, substantially less at KGI B and C and no chlorophyll at KGI A (Fig. 3C).

Fieldwork was carried out in summer 2014. Soil morphological description followed the guidelines of the Food and Agriculture Organization of the United Nations, and the pedons were classified using the World Reference Base system^51^. Samples were transported frozen to Germany and stored at a temperature of − 18 °C.

### Soil physics

Volumetric samples (100 cm^3^) for bulk density were taken from each horizon/depth increment with steel rings in three replicates. Bulk density [g cm^−3^] was gravimetrically determined including the correction by coarse material^52^. Bulk samples were air-dried and sieved < 2 mm. The grain size distribution (< 2 mm) of all samples was determined by combined sieving (2000–20 µm) and X-ray granulometry after using 1 M sodium metaphosphate (Na_4_P_2_O_7_) as a dispersant^53^.

### Pedochemical analyses and calculation of pedogenic oxide ratios and the Chemical Index of Alteration

Total nitrogen (N_t_) and soil organic carbon (SOC) were determined by thermal conductivity analysis after heat combustion (1150 °C) with a CNS-element analyzer (Elementar Vario EL III). AMS ^14^C dating was used to determine the age of the organic carbon of the samples with the highest SOC contents (Beta Analytic, Inc., USA). Soil pH_[H2O]_ and pH_[CaCl2]_ were determined potentiometrically in a 1:2.5 soil to water/0.01 M CaCl_2_ solution. Pedogenic Fe-(hydr-)oxides (Fe_d_) were extracted by dithionite-citrate-bicarbonate (DCB) solution^54^. Non- and poorly crystallized compounds of Fe (Fe_o_) and Al (Al_o_) were extracted by shaking 2.5 g of soil in 100 mL 0.2 M acid ammonium oxalate (pH 3) for 4 h in the dark^55^. The ratio between total Fe and pedogenic Fe-(hydr-)oxides (Fe_t_/Fe_d_) gives information on the iron release of Fe-bearing minerals, reflecting the intensity of weathering, whereas the ratio Fe_o_/Fe_d_ gives information on the degree of iron oxides crystallinity^56^. Major elements, including Fe (Fe_t_), were measured with a wavelength dispersive XRF device (PANanalytical PW 2400). Prior to preparation, the bulk samples (ratio Li-metaborate to soil 1:5) were ground with an agate mill for 10 min. The Chemical Index of Alteration (CIA) gives information on the ongoing chemical weathering and was calculated according to Nesbitt and Young^57^. The calculation was as follows [(Al_2_O_3_/Al_2_O_3_ + Na_2_O + CaO* + K_2_O)) × 100], where CaO* represents the amount of silicate-bound CaO.

### Nucleic acids extraction

The total genomic DNA of each sample was extracted in triplicates with the FastDNA^TM^ Spin Kit for soil (MO BIO Laboratories Inc., USA). In addition, a negative control without any template but the material and chemicals of the extraction kit was included. Sample replicates with very low DNA yields (Supplementary Table S1) were extracted in triplicates. These extraction triplicates of a sample replicate were merged and after reducing their volume to 50 µL by vacuum centrifugation ready for the following molecular biological work. DNA extracts were stored at − 20 °C and used as templates in the quantification of the bacterial 16S rRNA gene and high-throughput (HiSeq) sequencing.

### Illumina HiSeq-sequencing

Total genomic DNA extracts of each sample as well as an extraction negative control and a positive control (*Escherichia coli*) were sequenced using tagged 515F (5′-GTGCCAGCMGCCGCGGTAA-3′) and 806R (5′-GGACTACHVGGGTWTCTAAT-3′) primers^58^. The used cycler program and reaction mix were described by Meier et al.^59^. The sequencing was performed on an Illumina HiSeq (2 × 300 bp) by GATC Biotech AG, Germany.

### Bioinformatics and statistical analysis

The quality of raw sequencing data obtained by Illumina HiSeq (2 × 300 bp) was checked with FastQC^60^. The data were demultiplexed by using the *make.contigs* function in Mothur (version 1.39.5; pdiff = 2, bdiff = 1, and default setting for others^61^). According to the resulting report files, a filtering step was implemented to get fastq sequence identifiers for sequences with a minimum overlap of > 25 bases, maximum mismatches of < 5 bases and no ambigious bases. Next, these sequences were extracted with the *filterbyname.sh* function from BBTools^62^ from the raw paired-end fastq file. With QIIME1, sequence orientation was checked and corrected by using the *extract_barcodes.py* function and the primers were removed using the *awk* command^58^. DADA2 was used for filtering, dereplication, chimera check, sequence merge, and amplicon sequence variants (ASV) calling^63^. The output of DADA2 was taxonomically classified by using QIIME2^64^ and USEARCH^65^ with SILVA138^66^. ASVs with a relative abundance of < 0.01% in each sample were excluded from further analysis. For the processing and visualization of the obtained ASV table, R (vegan, gplots) and PAST4^67^ were used. Sample triplicates were merged by the mean value of their relative abundance before visualization of the sequencing data and before analysis of correlating environmental factors. The hierarchical clustering of the samples using the average linkage method was based on the Bray–Curtis dissimilarity. Demultiplexed raw sequencing data were submitted to the European Nucleotide Archive (http://www.ebi.ac.uk/ena) under the accession number PRJEB37594.

### Quantification of bacterial 16S rRNA gene copy numbers

Bacterial abundances were quantified using quantitative PCR (qPCR) and the 314F (5′-CCTACGGGAGGCAGCAG-3′) and 534R (5′-ATTACCGCGGCTGCTGG-3′) primers^68^. The used cycler program and reaction mix were described by Meier et al.^59^.

## Results

### Soil classification and soil properties

The soils KGI A, KGI B, and KGI C did not have properties to differentiate soil horizons and were classified as Hyperskeletic Cryosols. KGI D had distinct soil horizons and was classified as a Cambic Cryosol. Differences in vegetative cover and pedochemical properties were observed between the investigated soil profiles (Table 1, Supplementary Table S2).

**Table 1:** Major soil physical and soil chemical data, CIA and vegetation cover.

Soil	Depth (cm)	Vegetation (surface cover in %)	Horizon	BD^a)^ (g cm^−3^)	Sand (%)	Silt (%)	Clay (%)	N_t_ (%)	SOC (%)	pH		Pedogenic Ratios			CIA
			WRB^51^							H_2_O	CaCl_2_	Fe_o_/ Fe_d_	Fe_t_/Fe_d_	Al_o_*0.5 *Fe_o_	
***KGI A S 6° 09′ 991″, W 58° 28′ 007″, 38 m a.s.l***															
Hyperskeletic Cryosol	0–1	Bare soil	C	n.d	n.d	n.d	n.d	< 0.03	< 0.10	7.9	6.8	0.47	11.8	0.03	53.2
	0–10		C	1.08	50	32	18	< 0.03	< 0.10	8.3	7.5	0.77	12.5	0.05	53.7
	10–20		C	1.07	55	29	17	< 0.03	< 0.10	8.7	7.5	0.71	12.7	0.04	53.0
	20–40		C	n.d	52	28	19	< 0.03	< 0.10	8.9	7.8	0.54	10.2	0.06	51.2
***KGI B, S 6° 09′ 953″, W 58° 27′ 852″, 31 m a.s.l***															
Hyperskeletic Cryosol	0–1	*Usnea ant. (90), Deschampsia ant. (5), Colobanthus quit. (5); Total coverage 5*	C	0.98	n.d	n.d	n.d	< 0.03	< 0.10	7.4	6.6	0.21	12.6	0.02	49.9
	0–10		C	1.07	54	29	16	< 0.03	< 0.10	7.7	6.5	0.25	10.9	0.03	50.4
	10–20		C	n.d	62	23	15	< 0.03	< 0.10	8.3	7.2	0.19	12.2	0.02	49.9
	20–80		C	1.01	60	25	16	< 0.03	< 0.10	8.5	7.4	0.19	11.3	0.02	49.5
***KGI C, S 6° 09′ 947″, W 58° 27′ 862″, 40 m a.s.l***															
Hyperskeletic Cryosol	0–1	*Usnea ant. (70), Deschampsia ant. (10), Colobanthus quit. (10), Ochrolechia frigida (5), Mosses (5); Total coverage 80*	Ah	n.d	n.d	n.d	n.d	0.09	1.24	6.2	5.4	0.34	11.4	0.03	50.3
	0–10		AC	n.d	58	28	14	< 0.03	0.15	7.2	6.3	0.23	11.5	0.03	50.5
	10–20		C	n.d	60	27	14	< 0.03	< 0.10	8.1	7.0	0.31	10.9	0.04	50.2
	20–40		C	n.d	59	26	15	< 0.03	< 0.10	8.1	7.0	0.29	13.4	0.03	50.0
***KGI D, S 6° 09′ 976″, W 58° 28′ 260″, 54 m a.s.l***															
Cambic Cryosol	0–3	*Deschampsia ant. (50), Polytrichum spec. (40), Colobanthus quit. (5), Usnea ant. (5); Total coverage 100*	Ah	0.81	n.d	n.d	n.d	0.39	3.22	5.2	4.8	0.26	12.7	0.03	51.0
	3–15		Bw	0.97	62	31	8	0.03	0.24	6.3	5.1	0.16	12.5	0.02	50.9
	15–27		BC	0.98	62	27	11	< 0.03	< 0.10	7.3	5.9	0.16	14.7	0.02	49.3
	27–60		C	1.10	65	23	12	< 0.03	< 0.10	7.6	6.3	0.15	14.9	0.02	49.1

^a^Corrected by coarse material > 2 mm.

Regarding soil pH, similar trends were observed in the investigated soil profiles. The lowest pH_H20_/pH_CaCl2_ was found in the uppermost depth increment (A: 7.9/6.7; B: 7.4/6.5; C: 6.1/5.3; D: 5.1/4.8). With depth, pH_H20_/pH_CaCl2_ increased with the highest values in the lowermost depth increment (A: 8.8/7.7; B: 8.5/7.4; C: 8.05/6.99; D: 7.5/6.3). The vegetation cover was 0%, 5%, 80%, and 100% for KGI A, KGI B, KGI C, and KGI D, respectively. The vegetation cover at KGI B included *Usnea antarctica*, *Deschampsia antarctica*, and *Colobanthus quitensis*. In addition, no substantial accumulation of nitrogen (N_t_ < 0.03%) nor soil organic carbon (SOC < 0.05%) was observed. KGI C had higher N_t_ (0.09%) and SOC (1.24%) contents, and its surface was covered with vegetation comprising of *Usnea antarctica*, *Deschampsia antarctica*, *Colobanthus quitensis*, *Ochrolechia frigida*, and different mosses. The older and well developed soil, KGI D, showed distinct contents of nitrogen (0–3 cm: 0.39%; 3–15 cm: 0.03%), and of soil organic carbon (0–3 cm: 3.22%; 3–15 cm: 0.24%). The complete surface of KGI D was covered with *Deschampsia antarctica*, *Polytrichum spec*., *Colobanthus quitensis,* and *Usnea antarctica*. ^14^C dating showed that the humin fraction of soil organic matter in profile KGI C was formed after the melting of the glacier in 1979 (92.2% 1992–1995 cal AD (− 43 to − 46 cal BP); 3.2% 1957 cal AD (− 8 cal BP)), whereas the humin fraction of the upper 3 cm in profile KGI D is much older (95.4% 1954–1956 cal AD (− 5 to − 7 cal BP)) (Supplementary Table S3). The mainly volcanic substrate was not mirrored in the Al_o_ + ½Fe_o_ value, which is too low to indicate either andic (≥ 2%) or vitric (≥ 0.4%) properties. The Fe_o_/Fe_d_ and Fe_t_/Fe_d_ ratios, and the CIA did not show a clear direction of pedogenic or chemical weathering in KGI A, B, and C. In contrast, freshly formed Fe-(hydr-)oxides were indicated by the Fe_t_/Fe_d_ ratio (12.5–12.7) in the upper two horizons of KGI D. The Fe_o_/Fe_d_ ratio also shows a higher activity of Fe(hydr-)oxide formation in the upper horizons by a decreasing trend with depth in KGI D. The decreasing CIA with depth (51–49.1) designates initial silicate weathering processes combined with the dissolution of Ca, Na and K bearing minerals.

### Characterization and quantification of the microbial communities

High-throughput sequencing resulted in a mean of 577,306 reads per sample, ranging from 12,252 (KGI A 10–20 cm b) to 917,368 (KGI D 15–27 cm b) reads (Supplementary Tables S4 and S5). Rarefaction analysis revealed a sufficient sequencing depth in all samples for community analysis. The Shannon index showed a decreasing trend in diversity with depth across all profiles (Table 2), ranging between 6.2 and 5.3 in KGI A, 6.6–5.1 in KGI B, 6.8–5.7 in KGI C, and 6.7–5.8 in KGI D.

**Table 2:** Bacterial abundances and microbial diversity in four different soil profiles close to the Ecology Glacier, King George Island.

Sample	Bacterial 16 rRNA copies (gene copies g^−1^ soil)	Shannon's H	Evenness
KGI A 0–1	9.11 × 10^8^ ± 6.82 × 10^8^	6.20 ± 0.09	0.22 ± 0.03
KGI A 1–10	1.78 × 10^7^ ± 5.95 × 10^6^	6.05 ± 0.06	0.25 ± 0.01
KGI A 10–20	1.74 × 10^4^ ± 7.57 × 10^3^	5.31 ± 0.14	0.42 ± 0.03
KGI A 20–40	1.10 × 10^7^ ± 1.00 × 10^6^	5.85 ± 0.03	0.14 ± 0.00
KGI B 0–1	1.29 × 10^9^ ± 3.06 × 10^8^	6.46 ± 0.07	0.26 ± 0.01
KGI B 1–10	5.33 × 10^8^ ± 3.92 × 10^7^	6.61 ± 0.06	0.26 ± 0.02
KGI B 10–20	1.51 × 10^7^ ± 3.32 × 10^6^	5.40 ± 0.21	0.26 ± 0.12
KGI B 20–80	2.06 × 10^6^ ± 3.00 × 10^5^	5.16 ± 0.27	0.17 ± 0.10
KGI C 0–1	1.78 × 10^10^ ± 1.76 × 10^9^	6.85 ± 0.08	0.32 ± 0.01
KGI C 1–10	2.20 × 10^9^ ± 1.08 × 10^8^	6.79 ± 0.09	0.27 ± 0.01
KGI C 10–20	1.61 × 10^8^ ± 1.53 × 10^7^	5.74 ± 0.37	0.13 ± 0.02
KGI C 20–40	3.78 × 10^7^ ± 2.39 × 10^6^	5.79 ± 0.18	0.14 ± 0.02
KGI D 0–3	7.27 × 10^9^ ± 1.24 × 10^9^	6.78 ± 0.05	0.31 ± 0.02
KGI D 3–15	3.33 × 10^8^ ± 4.90 × 10^7^	6.23 ± 0.15	0.17 ± 0.01
KGI D 15–27	1.35 × 10^8^ ± 1.62 × 10^7^	5.87 ± 0.16	0.14 ± 0.02
KGI D 27–60	6.30 × 10^7^ ± 1.50 × 10^7^	6.03 ± 0.10	0.18 ± 0.01

**Figure 4:**
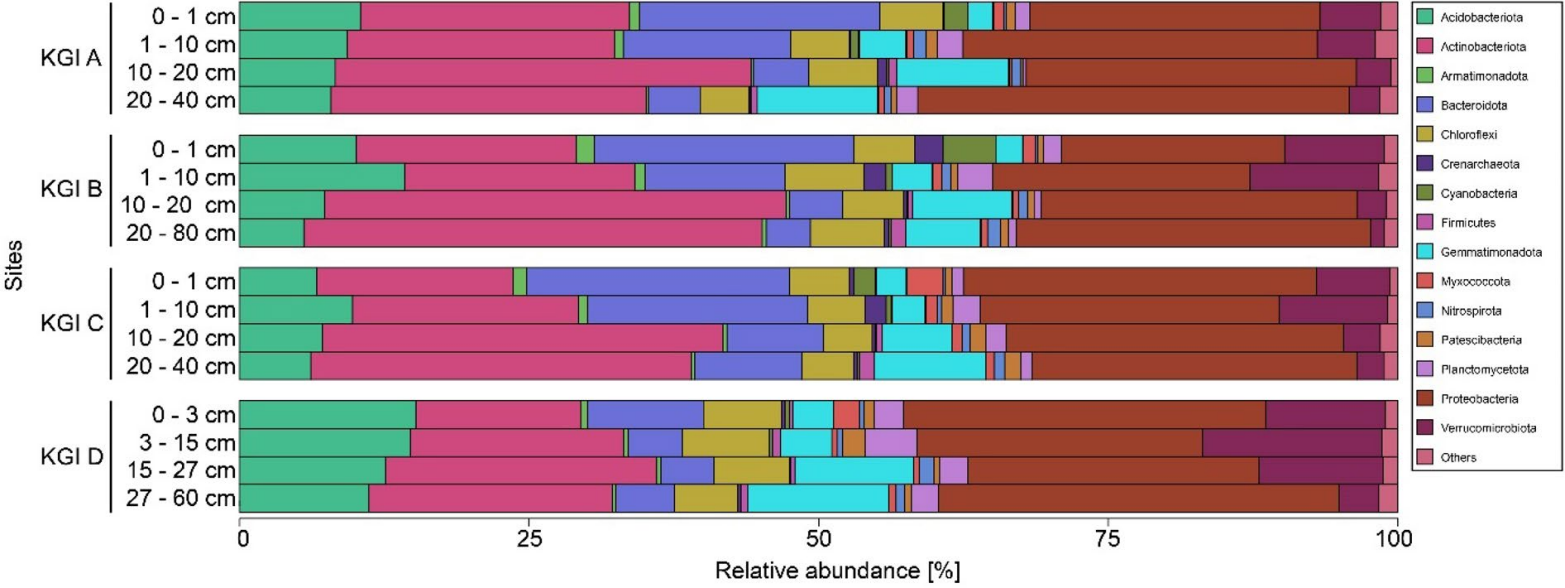
Relative abundances of phyla of three soil profiles (KGI A, KGI B, KGI C) in the recently deglaciated foreland of the Ecology glacier and one soil profile distal to the lateral moraine (KGI D) on King George Island, South Shetland Islands. Sample triplicates are merged. Only phyla with an abundance of at least 1% at a given site are presented. Less abundant phyla are summarized as “Others”.

The microbial communities were dominated by 9318 bacterial ASVs, which made up 96.9–99.9% of the observed reads in the investigated soils (Fig. 4). Looking at the total reads, a large fraction of ASVs is related the main phyla Proteobacteria (28.1%), Actinobacteriota (25.5%), Bacteroidota (10.6%), Acidobacteriota (9.7%), Verrucomicrobiota (6.2%), and Gemmatimonadota (6.1%). Comparing different profiles and soil depths, certain trends became visible. With depth, the relative abundances of Gemmatimonadota and Actinobacteriota increased, while the relative abundance of Bacteroidota and Verrucomicrobiota decreased.

Generally, KGI A, B, and C showed higher abundances of Actinobacteriota and Bacteroidota, whereas elevated relative abundances of Acidobacteriota and Verrucomibrobiota were observed in KGI D. 23 ASVs were associated with Archaea, which were made up mainly from Nitrososphaeraceae-related organisms within the phylum Crenarchaeota and showed relative abundances between 0 and 3%. Those Crenarchaeota showed their highest abundances in the upper 10 cm of KGI B and C, and comparably lower abundances in deeper soil layers across all profiles.

**Figure 5:**
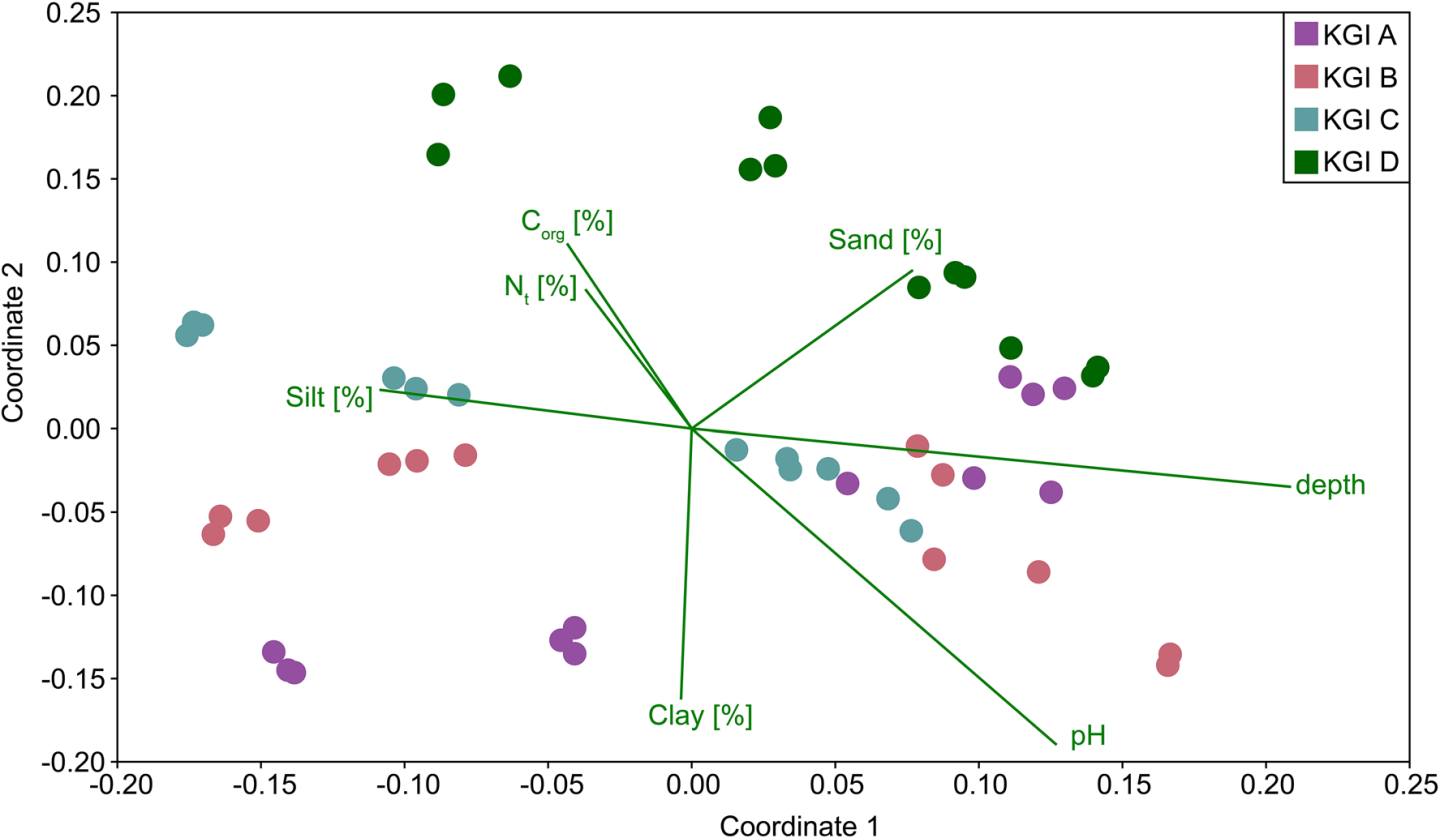
Non-metric multidimensional scaling plot comparing the microbial communities of three soil profiles in the foreland and one soil profile distal to the lateral moraine of the Ecology Glacier, King George Island, based on the Bray–Curtis dissimilarity. Environmental parameters were standardized using z-scores. The stress value was 0.11.

Soil microbial communities in the individual profiles tended to be different in the uppermost increment, but displayed an increasing similarity with depth, as shown by a NMDS (Fig. 5). Soil depth as well as pH, C_org,_ and N_t_ were the best factors to explain the respective microbial community structure of the investigated soils.

**Figure 6:**
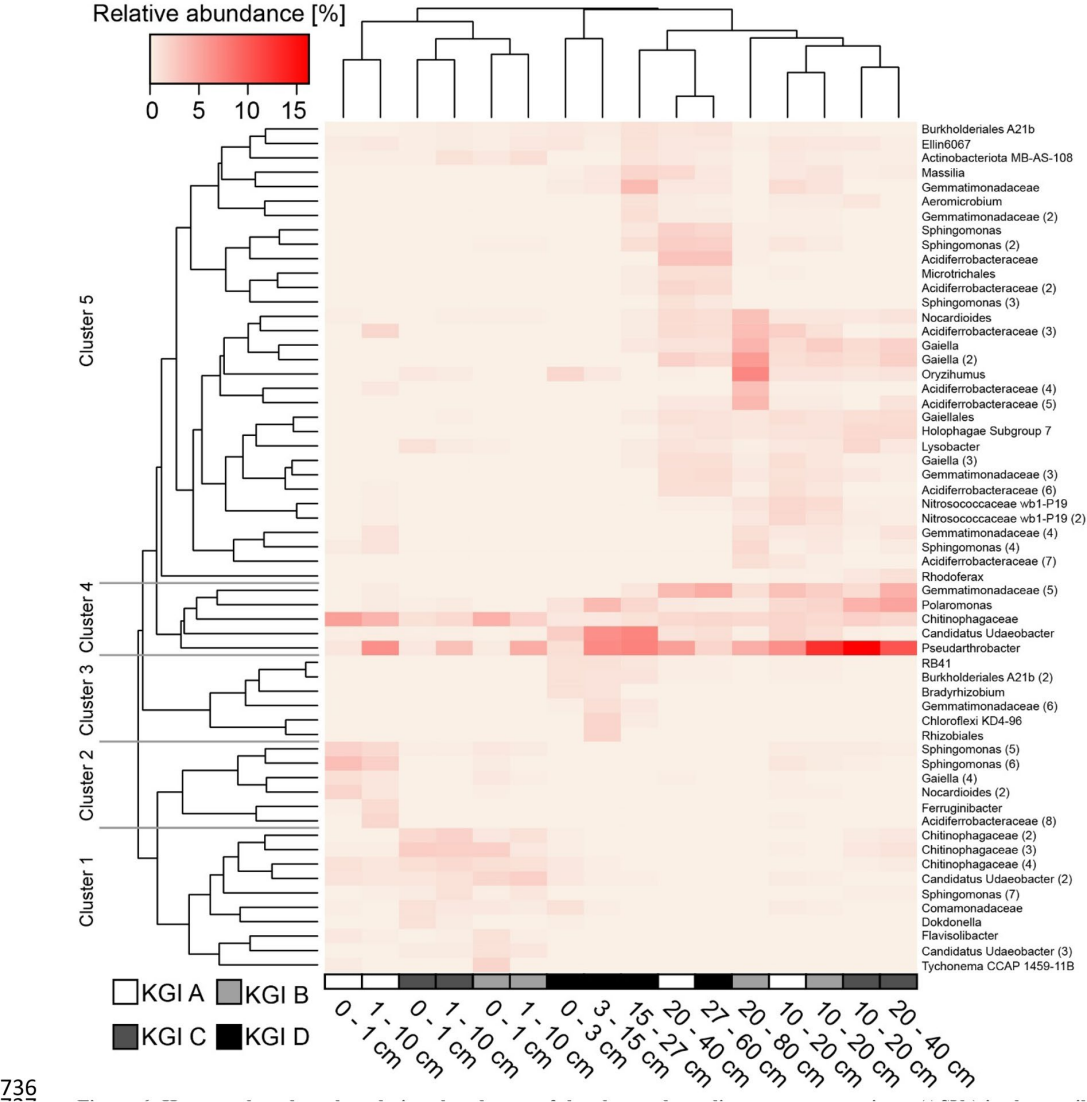
Heatmap based on the relative abundances of the observed amplicon sequence variants (ASVs) in three soil profiles (KGI A, KGI B, KGI C) in the recently deglaciated foreland of the Ecology glacier and one soil profile distal to the lateral moraine (KGI D) on King George Island, South Shetland Islands. Only ASVs with a relative abundance of at least 1.0% in a given sample are shown. Presented ASVs were clustered using average linkage hierarchical clustering. Samples were clustered based on the whole community using average linkage hierarchical clustering.

A cluster analysis of the investigated depth intervals, based on the Bray–Curtis dissimilarity, showed the distribution of abundant ASVs in the different sampling sites (Fig. 6). Samples were mainly clustered by the depth and to a lesser extent by the respective site. The analysis revealed five different clusters of abundant ASVs.

Cluster 1 included ten ASVs (Chitinophagaceae (2), Chitinophagaceae (3), Chitinophagaceae (4), Candidatus Udaeobacter (2), Sphingomonas (7), Comamonadaceae, Dokdonella, Flavisolibacter, Candidatus Udaeobacter (3), Tychonema CCAP 1459-11B) and was characteristic for the upper depth increments (0–10 cm) of the sites KGI B and KGI C. Cluster 2 was comprised of six ASVs (Sphingomonas (5), Sphingomonas (6), Gaiella (4), Nocardioides (2), Ferruginibacter, Acidiferrobacteraceae (8))) and characteristic for the upper depth increments (0–10 cm) of the site KGI A. Cluster 3 was comprised of six ASVs (RB41, Burkholderiales A21b (2), Bradyrhizobium, Gemmatimonadaceae (6), Chloroflexi KD4-96, Rhizobiales) and characteristic for the upper depth increments (0–10 cm) of site KGI D. The five ASVs associated within cluster 4 (Gemmatimonadaceae (5), Polaromonas, Chitinophagaceae, Candidatus Udaeobacter, Pseudarthrobacter) were not specific for any depth. Cluster 5 was comprised of 32 different ASVs (Burkholderiales A21b, Ellin6067, Actinobacteriota MB-AS-108, Massilia, Gemmatimonadaceae, Aeromicrobium, Gemmatimonadacae (2), Sphingomonas, Sphingomonas (2), Acidiferrobacteraceae, Microtrichales, Acidiferrobacteraceae (2), Sphingomonas (3), Nocardioides, Acidiferrobacteraceae (3), Gaiella, Gaiella (2), Oryzihumus, Acidiferrobacteraceae (4), Acidiferrobacteraceae (5), Gaiellales, Holophagae Subgroup 7, Lysobacter, Gaiella (3), Gemmatimonadaceae (3), Acidiferrobacteraceae (6), Nitrosococcaceae wb1-P19, Nitrosococcaceae wb1-P19 (2), Gemmatimonadaceae (4), Sphingomonas (4), Acidiferrobacteraceae (7), Rhodoferax) and was mainly connected to the lower depth increments (10/15–40/80 cm) of all sites.

Bacterial abundances determined by the quantification of the 16S rRNA gene showed similar trends across all profiles and varied in general between 10^4^ and 10^9^ copies g^−1^ soil (Table 2). KGI A (9.1 × 10^8^ copies g^−1^ soil), KGI B (1.3 × 10^9^ copies g^−1^ soil), KGI C (1.8 × 10^10^ copies g^−1^ soil), and KGI D (7.3 × 10^9^ copies g^−1^ soil) had the highest abundances in the uppermost soil layer. With depth, a substantial decrease in abundances was observed, resulting in the lowest abundances in KGI B (2.1 × 10^6^ copies g^−1^ soil), KGI C (3.8 × 10^7^ copies g^−1^ soil), and KGI D (6.3 × 10^7^ copies g^−1^ soil) in the lowermost soil layer. In KGI A, the lowest abundances with 1.74 × 10^4^ copies g^−1^ soil were found in a depth of 10–20 cm, before increasing to 1.1 × 10^7^ in the lowermost soil layer.

## Discussion

Glacier forelands provide an excellent opportunity to investigate initial soil formation and its pedochemical and biological drivers due to the transition from a glacial to a pedogenic geosystem. Over the last 50 years, the Ecology Glacier on King George Island has retreated 500 m inland^26^, exposing a large area to initial soil-forming processes, periglacial climate and the colonization of microbial pioneers. Our findings reveal differences in the soil-forming processes and their interaction with the microbial communities on decadal timescales compared to centennial to millennial timescales.

In accordance with observations in other Antarctic habitats^20,59,69^, the investigated soils were characterized by highly diverse microbial communities including Acidobacteriota, Bacteroidota, Verrucomicrobiota and especially high abundances of Actinobacteriota and Proteobacteria, which are known to thrive in recently deglaciated soils and facilitate a multitude of different phototrophic, photoheterotrophic and chemolithotrophic processes^11,70,71^. Differences between the sites with regards to the observed community compositions were found only in near-surface substrates in the upper 10 cm, while the microbial communities became less diverse and more similar with increasing depth across all investigated soil profiles. Multiple clusters of co-occurring and abundant ASVs for different sites and depths (Clusters 1, 2, 3, and 5) as well as a cluster of ubiquitous ASVs (Cluster 4) were observed. The most abundant ASVs in Cluster 4 were metabolically flexible organisms, such as ASVs related to Chitinophagaceae or *Polaromonas*. Several Chitinophagaceae-related ASVs were present in the investigated soils and especially abundant in the upper depth increments. Chitinophagaceae have been observed in Antarctic soils before^72,73^ and were described to play a role in the degradation of chitin and other soil organic compounds^74^. The degradation of organic compounds in the course of microbial respiration by Chitinophagaceae and other heterotrophic microorganisms could affect the soil pH and might enhance the chemical weathering close to the surface.

Additionally, a *Polaromonas*-associated ASV could be observed in all soils and depths (Cluster 4). These globally occurring organisms are able to survive in a dormant state^75^ and are, due to high levels of horizontal gene transfer, metabolically versatile^76^. These organisms are known to utilize a wide range of substrates such as H_2_^77^ or diverse organic compounds provided for instance by sea spray such as acetate, chloroacetate or octane^78^ and could be therefore a pioneer species in the recently exposed soil substrates in the foreland of the Ecology Glacier. In contrast to this cluster of frequently occurring ASVs, three different clusters consisted of abundant ASVs were found in the uppermost depth increments of the bare soil (KGI A, Cluster 2), slightly to moderately vegetated soil (KGI B and KGI C, Cluster 1), and fully vegetated soil (KGI D, Cluster 3). For instance, one important group within cluster 3 are ASVs related to Rhizobiales or Bradyrhizobium, which are known to be associated with the rhizosphere of plants, were particularly abundant in the fully vegetated site KGI D. These organisms are keyplayers for the fixation of nitrogen in soil ecosystems.

The differences in microbial community composition of the four study sites were also reflected in the microbial diversity of the near-surface depth increments, which increased slightly with vegetation coverage. Before lichens or vascular plants appear, abundant and diverse microbial communities are known to colonize recently exposed substrates^7,10^. These communities are dominated by photosynthetic, and heterotrophic N_2_-fixing bacteria^30^, resulting in an initial accumulation of labile carbon and nitrogen pools and play therefore an important role as pioneers for the further development of the fresh glacier forefield sediments. However, our dataset showed only low abundances of prokaryotic organisms most probably associated with phototrophic carbon fixation, such as the Cyanobacteria-related Tychonema. Low abundances of Cyanobacteria in recently deglaciated areas are not an uncommon observation in Antarctic soil environments^71,79^. In addition to the low abundances of Cyanobacteria, other bacterial groups might be involved in phototrophic carbon fixation, such as Chloroflexi^80^. Moreover, based on the on the amount of chloroplast-related sequences in our dataset, this process is at least partly facilitated by eukaryotic organisms such as algae in the early stage of soil development. Those microbial pioneers contribute to the stabilization and physical and chemical development of recently exposed substrates^81,82^, and initiate a cascade of crucial processes (e.g. carbon and nitrogen accumulation or bioweathering) that result in the formation of soils in which complex vegetation can grow^83^.

As mentioned above, microbial pioneer communities play an important role in initial soil formation. They alter the original soil environment; and are, in turn, influenced by ongoing pedogenic processes, succession, and plant colonization^82^. The site-specific microbial communities and the occurrence of defined clusters in the upper part of the soil profiles changed according to the vegetation coverage and potentially related soil properties such as the SOC or the soil pH.

Vegetation can influence the surrounding soil and its properties as well as the present soil microbiome in different ways, e.g. by releasing plant root exudates^84,85^, by plant litter input^34^ or by altering thermal and moisture retention of the soil^22^. To what extent the microbial communities in Antarctic soils are directly influenced by vegetation and vice versa is debated controversially^86–88^. As vegetation coverage increased, microbial communities shifted towards plant-related microorganisms in the foreland of the Damma Glacier in the Alps^11^. However, we could not observe similar effects on the microbial communities in the lower part of the investigated soils in the foreland of the Ecology Glacier. This is probably due to the lack of deeper roots of pioneer plants and the short time since plants colonized the foreland. The effect of plants on microbial community composition seemed to be limited to the upper part of the soils in the foreland of the Ecology Glacier since communities in depths > 10 cm were similar in all soil profiles regardless of plant coverage. Since more developed soils in the ice-free areas of Antarctica did not show mycorrhization, Boy et al.^34^ concluded that plants influence the colonized soil more by litter input than by direct transfer of photoassimilates to the surrounding soil. The input of plant litter leads to an increase of soil nitrogen and SOC contents especially in the upper centimeters of the soil. The succeeding decomposition of organic compounds in the course of microbial respiration could lead to a decrease of the pH value in the soil. In the upper and even in the lower part of the investigated soils, soil pH was altered by plant coverage, but shows only little influence on microbial community structure. In soil environments, pH usually is a significant attribute that shapes the present microbial community in favor of certain bacterial phyla^10,16,19,89^. Our results show that in the foreland of the Ecology Glacier, other vegetation-related properties, such as the SOC content, or soil moisture, and thermal retention, might influence the microbial communities close to the surface, not the pH value of the soils.

The SOC content has been shown to have a significant influence on microbial communities in cold habitats^8,10,11,16^. After the initial accumulation of labile carbon and nitrogen pools by microorganisms and the subsequent colonization of plants, the input of additional soil organic matter in the form of litter might sustain a richer heterotrophic community in the otherwise nutrient-poor environments of Antarctica. The presence of vegetation has been suggested to enhance the soil moisture and thermal retention of soils, thus reducing the severity of Antarctic conditions on the soil environment^22^. The soil moisture affects enzymatic and microbial activity^90^, the primary production^91^, and ultimately influences the microbial community structure in a variety of Antarctic habitats^89,92^. Another study showed that soil temperature affects microbial community composition and soil respiration^93^. In addition to the above-mentioned effects of plant colonization on SOC or soil pH, slightly higher and more stable moisture and temperature regimes due to the vegetation-related retention could lead to the differences in community compositions observed in the foreland of the Ecology Glacier, such as increased abundances of Verrucomicrobia-related species.

The present microbiome was influenced by the soil properties of the upper centimeters, such as the initial accumulation of SOC and nitrogen and the ongoing soil formation with its initial weathering processes and plant colonization. Conversely, the microbiome in deeper parts of the soil was affected by a variety of soil chemical parameters that change with depth (e.g. increase in soil pH, no quantifiable amounts of C and N), which explained a significant fraction of changes in the composition of the microbial community in the investigated soils and resulted in different, less abundant, and less diverse microbial communities. Eilers et al.^94^ compared several soil profiles in a forested montane watershed, where the most variable communities were located down to a depth of 10 cm and where less diverse and more similar microbial communities could be observed at depths > 10 cm regardless of the landscape position. They suggested that changes in soil properties with depth (e.g., pH, organic carbon quantity and quality, differences in temperature or moisture regimes) represent an ecological filter which makes it difficult for adapted surface-dwelling microorganisms to thrive, and causes a shift in the community composition in deeper soil horizons. Furthermore, changes in soil microstructure, induced e.g. by frequent freeze–thaw cycles and associated changes in pore spacing and nutrient contents have been related to shifts in microbial community compositions in soils from maritime Antarctica^59^. Meier et al. observed a change towards a lenticular microstructure below 20 cm depth, which was related to significant changes in the microbial community compositions. Some of the observed ASVs in deeper soils were *Acidiferrobacteraceae*-related organisms, which usually are associated with autotrophic lifestyles such as sulfur and iron oxidation, and have a broad range of possible substrates, such as ferrous iron, thiosulfate or ferric iron^95^. In initial soils on James Ross Island, Meier et al.^59^ found similar OTUs in the lower depth increments and connected those to mineral weathering in the course of microbial iron cycling. Cryoturbation, a process that would mix topsoil material with deeper soil horizons and vice versa, was reported to be influential for both abundance and diversity of bacterial communities in the foreland of the Ecology Glacier^39^. However, our results indicate that cryoturbation in these soils is of minor importance since in all soil horizons and at all study sites a clear differentiation with regard to the community structure with depth was evident.

Depth and soil properties influenced not only microbial diversity and community composition but also microbial abundances, which increased with vegetation cover and decreased substantially with soil depth. Exponentially decreasing microbial abundances and biomass with depth are a common observation in soil environments^94,96^. Although the investigated areas and soils are ice-free for just a few decades, the bacterial abundances were high (10^3^–10^10^ copies g^−1^ soil) showing similar trends across all investigated soils. Grzesiak et al.^97^ reported > 10^10^ counts per gram soil for the foreland of the Ecology Glacier. These high bacterial abundances are comparable to abundances observed in other parts of the Antarctic Peninsula^59^. A positive relationship between microbial abundances and vegetation as well as vegetation-related environmental factors (e.g. water content, organic carbon, and nitrogen content) was also observed by Yergeau et al.^86^. With increasing soil development along glacier forelands, defined by increasing carbon and nitrogen contents, decreasing pH, increasing vegetation coverage and increasing weathering ratios, we observed increasing microbial abundances which is consistent with other observations in cold environments^8,10^. Nevertheless, the relatively high abundances in the upper centimeters could also be influenced by algae and lichens such as *Usnea antarctica* and its chloroplasts.

The results show that on a decadal timescale after deglaciation, changes in microbial abundances, community compositions, and plant coverage are accompanied by lowering of the soil pH, and initial accumulation of SOC and nitrogen, which are the main soil-forming processes in the soils in the foreland of the Ecology Glacier. In contrast to these rather rapidly changing parameters, the quantifiable formation of pedogenic oxides and the increase in chemical weathering require much more time under the current climatic conditions of King George Island. The initial chemical weathering processes only became evident in the Cambic Cryosol of KGI D, which deglaciated before 1956. The main indication is the formation of Fe-(hydr-)oxides and a slight increase of the CIA at KGI D. On the other hand, the weathering related indices (Fe_t_/Fe_d_ and CIA) did not show a clear depth differentiation of pedogenic or weathering processes in the recently exposed soils (KGI A, KGI B, KGI C). Therefore, the chemical properties of the parent material remain almost unaltered.

Generally considered, weathering efficiency is strongly dependent on the ambient temperature^98^. Compared to temperate ecosystems, soils in high latitudes form over longer periods of time^99^. Despite the low metabolic activity, soil organisms such as bacteria, fungi, and nematodes promote soil-forming processes in maritime Antarctica^100^ by driving the nitrogen and carbon cycle^101–103^, and affecting weathering processes in Antarctic soils^104^. By performing enzymatically catalysed reactions, processes reducing the pH and the production of complexing agents^3,98, 105^ microorganisms are able to substantially promote weathering processes^82^. The biological weathering of rock material is a crucial process that maintains a continuous supply of inorganic nutrients for prokaryotic and eukaryotic life in barren environments^106,107^ and might be of major importance for the ongoing ecological succession towards more complex communities in recently exposed substrates. Certain prokaryotic genera present in the investigated soils, such as *Polaromonas* or *Massilia*, were associated with mineral weathering in the past^108^. Frey et al.^109^ showed that such microorganisms could enhance elemental release from granite by colonizing rock surfaces and lowering the ambient pH by secreting organic acids and hydrogen cyanide for instance. This process may be also responsible for the lowering of the pH values in the upper two depth increments of the bare soil KGI A. Subsequently, the respiration of organic matter originating from plant litter by an active, diverse and abundant heterotrophic community including for example Chitinophagaceae could further decrease the soil pH, and thus impact weathering rates especially over longer timescales.

## Conclusions

This study contributes to a better understanding of the interrelation between microbial communities and soil-forming processes in recently deglaciated Antarctic soil substrates and the timescales required for such processes. We found highly diverse communities of microbial pioneers and plants, particularly in the upper part of soils, formed in the same substrate in the foreland deglaciated after 1979 of the Ecology Glacier and distal to its lateral moraine (deglaciated before 1956). In the upper depth increments, differences in the soil chemical and microbiological properties were found even between the three sites in the foreland (KGI A, B, C), which became ice-free at the same time. Soil pH and SOC depended on the vegetation coverage of the respective site and especially the soil pH in the vegetated sites could be impacted by microbial degradation of plant litter. The lowering of the soil pH in the bare soil, however, may be explained by more active Chitinophagaceae and other potential heterotrophs, and the degradation of organic material of microbial origin, such as chitin from fungi.

Soil depth represents a variety of changes in the environment such as the increase in soil pH or the decrease in organic carbon contents and was the strongest determining factor explaining the decrease in microbial diversity and abundances. The microbial communities were similar at all sites in > 10 cm, regardless of their exposure age after deglaciation. This means that cryoturbation processes may not have played a major role since the deglaciation, otherwise we would not have obtained clear depth functions of soil properties such as SOC and N_t_ content, or additionally of the Fe_d_/Fe_t_ ratio and the CIA at the oldest site KGI D.

On a decadal timescale after deglaciation, changes in soil pH, and initial accumulation of soil carbon and nitrogen were the main soil-forming processes, which were accompanied by changes in microbial abundances, community compositions, and plant cover. On a centennial to a millennial timescale after deglaciation, quantifiable silicate weathering and formation of pedogenic (hydr-)oxides could be observed. The cold climate of Antarctica slows down microbial weathering processes and soil formation rates on recently exposed sediments. However, we conclude that prokaryotic microorganisms initiate measurable changes of soil properties such as pH at a very early stage (within decades) before the soil surface is colonized by pioneer plants or soil horizons other than C horizons are detectable, and thereby promote weathering processes. To further verify our conclusions and illuminate the microbial processes driving soil formation, in future studies multiple comparable setups (freshly deglaciated material vs. older, more matured soil close to the foreland) could be studied and include metagenomic and metatranscriptomic analyses.

## Supplementary Information

Supplementary Tables.

Supplementary Table S5.

## Data Availability

Demultiplexed raw sequencing data were submitted to the European Nucleotide Archive (http://www.ebi.ac.uk/ena, last access: 2 June 2020) under accession number PRJEB37594.
