# Supplementary material for: Influence of prokaryotic microorganisms on initial soil formation along a glacier forefield on King George Island, maritime Antarctica
Source: Sci Rep. 2021 Jun 23;11:13135. doi: 10.1038/s41598-021-92205-z (PMC8222374; doi:10.1038/s41598-021-92205-z)
Supplement: Supplementary file 1 — Supplementary Tables. [file 41598_2021_92205_MOESM1_ESM.pdf]

Influence of prokaryotic microorganisms on initial soil formation along a glacier forefield on King George Island, maritime Antarctica

Patryk Krauze[1*], Dirk Wagner[1,2], Sizhong Yang[1], Diogo Spinola[3,4] and Peter Kühn[3]

[1]GFZ, German Research Centre for Geosciences, Helmholtz Centre Potsdam, Section Geomicrobiology, 14473 Potsdam, Germany

[2]Institute of Geosciences, University of Potsdam, 14476 Potsdam, Germany

[3]Department of Geosciences, Research Area Geography, Laboratory of Soil Science and Geoecology, Eberhard Karls University Tübingen, 72070 Tübingen, Germany

[4]Present address: Department of Chemistry and Biochemistry, University of Alaska Fairbanks, 99775-6160 Fairbanks, USA

**Corresponding author*: Patryk Krauze (pkrauze@gfz-potsdam.de)

Table S1: Amount of soil used for DNA extraction and resulting DNA concentrations.

sample	soil used for extraction [mg]	DNA concentration [ng/µl]
KGI_A_0_1_a	557	7.13
KGI_A_0_1_b	550	8.2
KGI_A_0_1_c	530	6.87
KGI_A_1_10_a	510	0.121
KGI_A_1_10_b	564	0.213
KGI_A_1_10_c	585	0.103
KGI_A_10_20_a	1560	n. d.
KGI_A_10_20_b	1585	n. d.
KGI_A_10_20_c	1631	n. d.
KGI_A_20_40_a	1510	0.4
KGI_A_20_40_b	1614	0.511
KGI_A_20_40_c	1574	0.376
KGI_B_0_1_a	505	13.3
KGI_B_0_1_b	494	10.1
KGI_B_0_1_c	541	9.13
KGI_B_1_10_a	590	4.61
KGI_B_1_10_b	590	4.83
KGI_B_1_10_c	549	4.11
KGI_B_10_20_a	592	0.09
KGI_B_10_20_b	538	0.04
KGI_B_10_20_c	529	0.08
KGI_B_20_80_a	1679	0.07
KGI_B_20_80_b	1665	0.09
KGI_B_20_80_c	1639	0.04
KGI_C_0_1_a	485	78.4
KGI_C_0_1_b	500	69.6
KGI_C_0_1_c	550	61.2
KGI_C_1_10_a	548	18.5
KGI_C_1_10_b	544	16.5
KGI_C_1_10_c	553	12.4
KGI_C_10_20_a	530	0.8
KGI_C_10_20_b	570	1.17
KGI_C_10_20_c	565	1.13
KGI_C_20_40_a	550	0.273
KGI_C_20_40_b	570	0.428
KGI_C_20_40_c	562	0.101
KGI_D_0_3_a	552	49.6
KGI_D_0_3_b	505	49.4
KGI_D_0_3_c	585	22.6
KGI_D_3_15_a	560	5.46
KGI_D_3_15_b	514	3.87
KGI_D_3_15_c	512	4.97
KGI_D_15_27_a	562	1.35
KGI_D_15_27_b	561	1.27
KGI_D_15_27_c	586	1.37

KGI_D_27_60_a	599	0.322
KGI_D_27_60_b	550	0.337
KGI_D_27_60_c	548	0.501

Table S2: Major elements by XRF of four soil profiles from King Georges Island. Antarctica. All data given in weight percent. LOI (loss on ignition) determined at 1000°C for one hour. For location of the profiles. see Table 1.

Profile	Depth	SiO_2	Al_2O_3	Fe_2O_3	MnO	MgO	CaO	Na_2O	K_2O	TiO_2	P_2O_5	LOI	Sum
	[cm]		%										
KGI A													
	0-1	49.43	0.93	19.39	8.53	0.20	3.38	5.85	3.13	1.16	0.24	7.46	99.8
	1-10	48.98	0.94	19.30	8.48	0.20	3.15	5.72	3.00	1.18	0.25	8.39	99.7
	10-20	49.25	0.91	19.26	8.26	0.19	3.03	5.95	3.04	1.19	0.25	8.30	99.7
	20-40	49.73	0.93	19.12	8.54	0.20	3.11	6.26	3.41	1.16	0.24	7.12	99.9
KGI B													
	0-1	49.80	0.95	18.77	8.72	0.21	3.26	6.31	3.74	1.14	0.27	6.28	99.5
	1-10	49.72	0.94	18.95	8.71	0.21	3.29	6.29	3.65	1.12	0.26	6.87	100.
	10-20	49.92	0.93	18.85	8.69	0.21	3.18	6.34	3.76	1.15	0.27	6.36	99.8
	20-80	49.66	0.94	18.83	8.73	0.20	3.25	6.51	3.71	1.14	0.26	6.43	99.8
KGI C													
	0-1	48.43	0.85	18.65	8.30	0.20	3.47	6.23	3.62	1.05	0.27	8.73	99.9
	1-10	49.45	0.94	18.91	8.76	0.20	3.22	6.24	3.63	1.09	0.28	6.88	99.7
	10-20	49.66	0.95	18.91	8.89	0.21	3.09	6.29	3.69	1.13	0.29	6.68	99.9
	20-40	49.73	0.94	18.90	8.67	0.19	3.10	6.30	3.73	1.17	0.29	6.35	99.5
KGI D	0-3	44.39	0.79	17.47	7.75	0.17	3.40	5.88	2.98	1.12	1.04	14.6	99.7
	3-15	48.84	0.82	19.22	8.06	0.18	3.54	6.30	3.53	1.19	0.35	7.84	100.
	15-27	49.85	0.80	19.28	7.81	0.19	3.50	6.80	3.79	1.15	0.21	6.54	100.
	27-60	49.24	0.82	19.30	8.04	0.20	3.56	7.01	3.71	1.08	0.19	6.49	99.8

Table S3: Radiocarbon dating results obtained on the humin fraction of soil organic matter from two sites at the Ecology Glacier. King George Island.

Profile	Depth [cm]	Horizon	Material	pMC [%]	δ13C (‰)	cal BP (1σ)	cal CE (1σ)	Lab-Nr.
KGI C	0 – 1	Ah	soil organic matter. humin fraction (alkali soluble organics)	112.55 ± 0.42	-26.1	-44 to -45 cal BP	1993 - 1994	Beta-570459
KGI D	0 - 3	Ah	soil organic matter. humin fraction (alkali soluble organics)	100.62 ± 0.38	-27.9	-5 to -6 cal BP	1954 - 1955	Beta-570458

Table S4: Number of sequencing reads after each processing step.

sample	input	filtered	denoised	merged	Non-chimera	0.01% cutoff
	number of reads					
KGI_A_0_1_a	823555	760184	744842	728869	722825	706168
KGI_A_0_1_b	416652	386897	378089	368092	365389	359124
KGI_A_0_1_c	923881	848435	824280	799372	786254	772324
KGI_A_1_10_a	473836	436670	427179	416676	413400	410050
KGI_A_1_10_b	234950	218204	212241	204825	203788	201502
KGI_A_1_10_c	352840	330275	323230	316343	314148	311452
KGI_A_10-20_a	36923	33865	29718	26130	25958	25922
KGI_A_10-20_b	18966	17275	14209	12268	12259	12252
KGI_A_10-20_c	25875	23729	19871	17313	17308	17300

KGI_A_20_40_a	905510	839764	828694	814373	808267	790148
KGI_A_20_40_b	846168	790425	779458	764252	758482	737591
KGI_A_20_40_c	893543	830439	821643	808578	801514	778446
KGI_B_0_1_a	802491	738909	726606	711994	705038	683957
KGI_B_0_1_b	767042	707072	685728	660207	650385	634947
KGI_B_0_1_c	776140	717207	691876	662511	644483	630913
KGI_B_1-10_a	870220	801229	775510	742168	726536	700009
KGI_B_1-10_b	763113	708362	698110	683584	679585	655095
KGI_B_1-10_c	984339	904070	867753	821445	790478	763807
KGI_B_10_20_a	902645	835484	829409	818332	809608	796113
KGI_B_10_20_b	50853	47043	41987	37904	37729	37613
KGI_B_10_20_c	45144	41598	36756	32746	32618	32557
KGI_B_20_80_a	797077	739614	734477	727607	713996	706767
KGI_B_20_80_b	666167	617867	613646	605338	594001	587915
KGI_B_20_80_c	59859	55236	50726	47120	46730	46649
KGI_C_0_1_a	748549	692351	673800	648601	635472	605915
KGI_C_0_1_b	896152	828214	784842	728938	687678	661043
KGI_C_0_1_c	780292	724237	701902	672607	650189	619970
KGI_C_1_10_a	1072129	979738	941569	893583	866371	830430
KGI_C_1_10_b	796453	734715	714128	686413	675197	651021
KGI_C_1_10_c	945868	879402	852158	818052	799307	765149
KGI_C_10_20_a	914463	847130	837834	821943	814309	788037
KGI_C_10_20_b	724047	663724	657595	646501	642849	625122
KGI_C_10_20_c	379777	348578	342785	334296	331763	325740
KGI_C_20_40_a	805325	743983	737314	727505	720592	705527
KGI_C_20_40_b	883724	818626	808175	789997	782377	762057
KGI_C_20_40_c	651443	603352	595920	583664	578353	564023
KGI_D_0_3_a	779880	715854	698512	675201	664504	626027
KGI_D_0_3_b	787246	723529	685098	643359	617163	586001
KGI_D_0_3_c	898392	826444	770393	701924	643856	619137
KGI_D_3_15_a	815335	754410	739659	718535	709893	677501
KGI_D_3_15_b	950935	871121	850341	820666	806893	766920
KGI_D_3_15_c	930601	858951	806053	732072	650658	627047
KGI_D_15_27_a	913147	851119	839448	822669	812036	784157
KGI_D_15_27_b	1074041	989909	980950	964946	953467	917368
KGI_D_15_27_c	869976	799507	791364	777160	770119	749250
KGI_D_27_60_a	740686	687100	681491	671687	666209	645648
KGI_D_27_60_b	768476	714698	707202	695134	689840	671215
KGI_D_27_60_c	852927	790621	782334	769492	763600	737767
Negative control	11045	9999	9955	9899	9899	9819
Positive control	651120	590140	589778	585268	585268	585250
